# Implementation of remote consulting in UK primary care following the COVID-19 pandemic: a mixed-methods longitudinal study

**DOI:** 10.3399/BJGP.2020.0948

**Published:** 2021-02-09

**Authors:** Mairead Murphy, Lauren J Scott, Chris Salisbury, Andrew Turner, Anne Scott, Rachel Denholm, Rhys Lewis, Geeta Iyer, John Macleod, Jeremy Horwood

**Affiliations:** Centre for Academic Primary Care (CAPC), University of Bristol, Bristol Medical School, Bristol.; National Institute for Health Research Applied Research Collaboration West (NIHR ARC West), University Hospitals Bristol and Weston NHS Foundation Trust, Bristol; CAPC, University of Bristol, Bristol Medical School, Bristol.; National Institute for Health Research Applied Research Collaboration West (NIHR ARC West), University Hospitals Bristol and Weston NHS Foundation Trust, Bristol; CAPC, University of Bristol, Bristol Medical School, Bristol.; National Institute for Health Research Applied Research Collaboration West (NIHR ARC West), University Hospitals Bristol and Weston NHS Foundation Trust, Bristol; CAPC, University of Bristol, Bristol Medical School, Bristol.; Centre for Academic Primary Care (CAPC), University of Bristol, Bristol Medical School, Bristol.; NIHR Bristol Biomedical Research Centre, University of Bristol; CAPC, University of Bristol, Bristol.; One Care, Whitchurch, Bristol.; North Somerset and South Gloucestershire Clinical Commissioning Group, Bristol.; National Institute for Health Research Applied Research Collaboration West (NIHR ARC West), University Hospitals Bristol and Weston NHS Foundation Trust, Bristol; CAPC, University of Bristol, Bristol Medical School, Bristol.; National Institute for Health Research Applied Research Collaboration West (NIHR ARC West), University Hospitals Bristol and Weston NHS Foundation Trust, Bristol; CAPC, University of Bristol, Bristol Medical School, Bristol.

**Keywords:** general practitioners, online consultation, remote consultation, telephone consultation, triage

## Abstract

**Background:**

To reduce contagion of COVID-19, in March 2020 UK general practices implemented predominantly remote consulting via telephone, video, or online consultation platforms.

**Aim:**

To investigate the rapid implementation of remote consulting and explore impact over the initial months of the COVID-19 pandemic.

**Design and setting:**

Mixed-methods study in 21 general practices in Bristol, North Somerset and South Gloucestershire.

**Method:**

Longitudinal observational quantitative analysis compared volume and type of consultation in April to July 2020 with April to July 2019. Negative binomial models were used to identify if changes differed among different groups of patients. Qualitative data from 87 longitudinal interviews with practice staff in four rounds investigated practices’ experience of the move to remote consulting, challenges faced, and solutions. A thematic analysis utilised Normalisation Process Theory.

**Results:**

There was universal consensus that remote consulting was necessary. This drove a rapid change to 90% remote GP consulting (46% for nurses) by April 2020. Consultation rates reduced in April to July 2020 compared to 2019; GPs and nurses maintained a focus on older patients, shielding patients, and patients with poor mental health. Telephone consulting was sufficient for many patient problems, video consulting was used more rarely, and was less essential as lockdown eased. SMS-messaging increased more than three-fold. GPs were concerned about increased clinical risk and some had difficulties setting thresholds for seeing patients face-to-face as lockdown eased.

**Conclusion:**

The shift to remote consulting was successful and a focus maintained on vulnerable patients. It was driven by the imperative to reduce contagion and may have risks; post-pandemic, the model will need adjustment.

## INTRODUCTION

In the last decade, remote primary care consultations, conducted by telephone, video, or through asynchronous text-based GP–patient communication via email or an online portal (e-consultation) have become more prevalent. In Denmark, GP telephone triage in out-of-hours care and doctor–patient emails are standard practice.^1^ In the US, Kaiser Permanente offer secure GP–patient email communication and routine telephone/video consultations.^2^ In the UK, telephone consulting is widespread but, prior to the COVID-19 pandemic, video consultations were very rare^3^^,^^4^ and take up of e-consultations were low^5^ but increasing.^6^

The NHS Long Term Plan committed practices to offer e-consultations from April 2020 and video from April 2021.^7^ The COVID-19 pandemic required accelerated adoption of these tools.^8^ In March 2020, to reduce contagion, the UK government instructed general practice to conduct all consultations remotely unless there was urgent need otherwise.^9^ The public were instructed to ‘Stay at home, protect the NHS, and save lives’.^10^ Patients at high risk of severe COVID-19 were advised to ‘shield’, avoiding all but essential contact. Telephone or e-consultation triage models were introduced,^11^ with most triaged consultations done via telephone and/or video.^12^ The market in the UK was ready for this change. Companies that offer video and e-consultations have been expanding since 2018^6^ and accuRx SMS Chain, which enables GPs to send SMS messages direct from the patient record, was operational in 50% of GP practices in England by February 2020.^13^ In March 2020, technology companies offered remote consulting technologies at low or zero cost.^14^^,^^15^

The move to remote consulting has been successful in implementation,^16^^,^^17^ but concerns about collateral effects have been raised.^18^ There has been little empirical study on the impact. The aim of this study was to investigate the impact of the rapid implementation of remote consultations in March 2020 on the delivery of patient care and explore how this changed during the first 4 months of the COVID-19 pandemic. The research questions were:

1. how did the volume and type of consultations and communications with patients change over the period April 2020 to July 2020 and how did this differ from the same period in 2019;
2. did changes in consulting rates or types of consultation provided differ across patient groups; and
3. how did primary care clinicians and managers deal with the rapid implementation of remote consulting.

**Table:** How this fits in

The COVID-19 pandemic has rapidly accelerated a move to remote consulting (telephone, video, and online) in general practice. In this mixed-methods longitudinal study, the authors found that this shift to remote consulting had some benefits but was driven by necessity and helped by low consultation volumes in March and April 2020. Despite a drop in consultations overall, contact rates increased or stayed the same for patients who were older, shielding, or had poor mental health. As consultation rates returned to normal by July 2020 and patients began to consult with more complex problems, GPs found remote management can be more time-consuming, clinically challenging, and less satisfying. The appropriate role of remote consulting in future primary care service delivery remains unclear.

## METHOD

The authors conducted a mixed-methods study in 21 general practices in Bristol, North Somerset and South Gloucestershire Clinical Commissioning Group (BNSSG CCG).

Practices were provided with study information by BNSSG CCG. Practices returned expressions of interest and 21 practices were recruited (25% of BNSSG practices). Practices were selected to include maximum variation on practice size, deprivation, location, and ethnicity mix (see Supplementary Table S1). All practices used the EMIS patient records system. Pre-pandemic plans for the roll-out of an algorithm-based e-consultation platform across the CCG were implemented during the study.

### Quantitative methods

A longitudinal analysis of consultations in the 21 practices was conducted to address research questions 1 and 2. One Care (https://onecare.org.uk), the GP federation in BNSSG CCG, extracted data on: demographic and clinical characteristics of patients registered in July 2020; consultations these patients had with clinicians from January 2019 to July 2020; and clinical codes entered in February to July 2019 and February to July 2020. Characteristics extracted included age, ethnicity, deprivation, sex, shielding status, and mental health status. Sex, age, deprivation, and shielding status (as of July 2020) were extracted from patients’ records. Poor mental health was defined as either severe mental illness, diagnosed depression, or prescribed antidepressants (excluding tricyclics) in the 3 months prior to July 2020. Ethnicity was derived from the patient record (see Supplementary Box S1).^19^

The authors defined consultations as two-way interactions between patients and GPs or nurses and/or paramedics. Consultations were excluded if added by administrators. This meant that, if an e-consultation added by an administrator was managed by the GP through, for example, a telephone call, this is registered as a telephone consultation, but the e-consultation is not within the present study data. Administrative tasks performed by clinicians were also excluded. A combination of consultation details and clinical codes were used to identify consultation types (face-to-face, home visit, telephone, video, and e-consultations) and staff type (GP and nurse or paramedic). To examine changes in clinician–patient communications outside of consultations, the authors separately identified SMS messages sent from clinicians to patients (see Supplementary Figure S1).

#### Descriptive statistics

Consultations and SMS message rates are reported per 1000 patients. The authors adjusted the patient characteristic data extracted in July 2020 by historical practice list sizes to calculate monthly list sizes.^20^

#### Multilevel model

Changes in consultation rates between April to July 2019 and April to July 2020 were investigated using negative binomial models. The model outcomes were: all consultations, remote consultations (telephone, video, or e-consultation), and in-person consultations (face-to-face or home visit). Only consultations were included in the models, not SMS messages. Incidence rate ratios (IRRs) and 95% confidence intervals (CIs) are reported for consulting rates in 2020 compared to 2019. Models were developed for each outcome and staff type (GP and nurse or paramedic). Consultation year was fitted as a fixed effect, GP practice as a random effect, and adjusted practice list size as the offset. Fixed effects for each of the covariates (age, sex, ethnicity, Index of Multiple Deprivation [IMD] quintile, shielding status, and mental health status), and an interaction term between each covariate and consultation year, were fitted separately to each model to explore differences in the outcomes across patient characteristics. Model validity was checked using standard methods; outliers which disrupted model fit were removed.

### Qualitative methods

Longitudinal interviews were conducted with a purposeful sample of practice staff based on job role. Interviews used a flexible topic guide (see Supplementary Box S2) to investigate how practices dealt with the implementation of remote consulting, challenges faced, and solutions developed. There were 87 interviews conducted in four rounds between 13 May to 29 July 2020, with 41 participants: 21 GPs, 11 practice managers, and nine senior nurses and/or advanced nurse practitioners (see Supplementary Table S1).

Interviews were audiorecorded and summarised into a framework based on the topic guide. Towards the end of each round, the authors read across this framework (within each topic) and down (within each interview) to identify themes relevant to that round and produced a rapid intelligence report. The authors presented each report at a CCG primary care COVID-19 strategy group meeting, receiving feedback on key issues to consider in the next round.^21^ The audio files were transcribed and imported into NVivo for analysis.

Normalisation Process Theory (NPT)^22^ was used to structure the analysis of the implementation of remote consulting. NPT proposes that implementation of a complex intervention is dependent on staff fulfilling four criteria:

1. coherence (making sense of the reasons for remote consulting);
2. cognitive participation (buy-in to remote consulting);
3. collective action (putting remote consulting into action); and
4. reflexive monitoring (appraising the consequences of the move to remote consulting).

Four authors established an initial coding framework within the four NPT constructs (see Supplementary Box S3). Three of these authors double-coded six interviews (two each) to ensure a coding consensus and maximise rigour.^23^ Then, all data was coded relating to remote consulting into the final framework.

## RESULTS

### Change in consultation numbers

**Figure 1.**
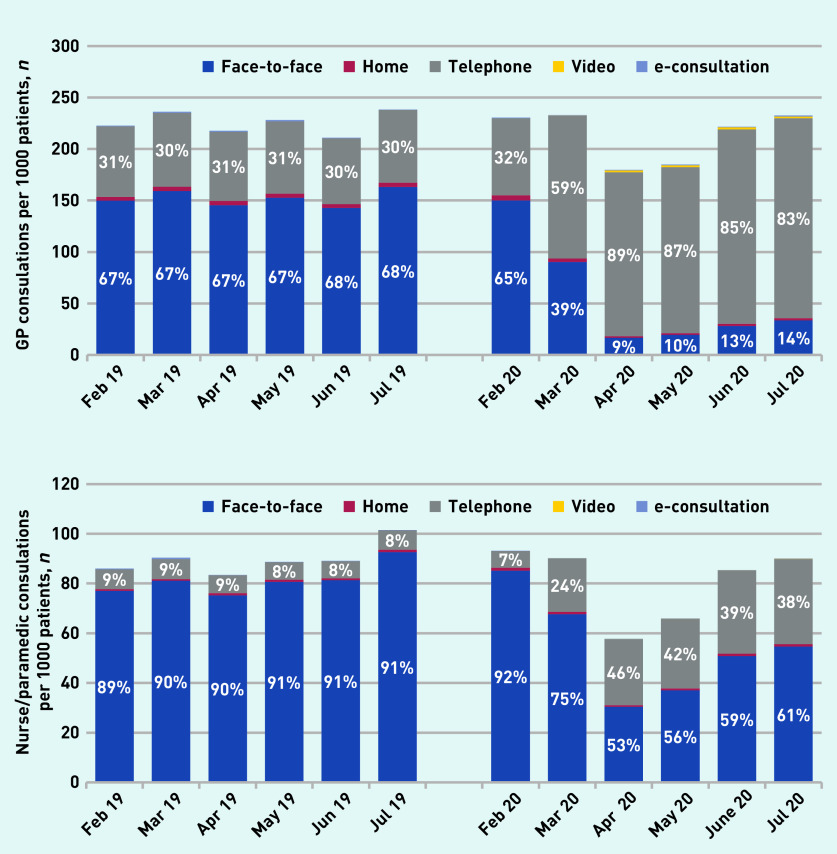
***GP and nurse/paramedic consultations per 1000 registered patients, February to July 2019 and February to July 2020. Between 97% and 99% of consultations each month were face-to-face or telephone. Monthly percentages for home visits, e-consultations, and video (not shown) varied between 1% to 3% across all three types.***

The quantitative findings are based on 350 966 registered patients in 21 practices (Table 1). In April 2019 there were 218 GP consultations per 1000 registered patients; 31% were by telephone, and no video consultations were recorded (Figure 1). In April 2020 this had reduced to 180 GP consultations per 1000 registered patients; 89% by telephone and 1% coded as video, increasing to 3% for patients aged >85 years (although GP coding practices may mean that some video consultations were coded as telephone consultations). Less than 1% of consultations coded by GPs were e-consultations. Consultation volumes increased by June/July 2020 back to similar levels as seen in June/July 2019.

**Table 1.** Characteristics of all patients registered in participating practices in July 2019

	**Registered patients, *n***	**%**
**Age, years**		
0–4	18 685	5.32
5–17	46 822	13.34
18–49	158 993	45.30
50–69	77 952	22.21
70–84	35 875	10.22
≥85	12 639	3.60

**Sex**		
Male	175 952	50.13
Female	175 009	49.86
Missing	5	<0.01

**IMD quintile**		
1 (most deprived)	71 378	20.34
2	55 002	15.67
3	53 129	15.14
4	73 119	20.83
5 (least deprived)	96 608	27.53
Missing	1730	0.49

**Ethnicity**		
White	228 624	65.14
Asian/Asian British	11 774	3.35
Black/African/Caribbean/Black British	12 234	3.49
Mixed/Multiple ethnic groups	5368	1.53
Other	1210	0.34
Missing	91 756	26.14

**Mental health**		
Good	318 329	90.70
Poor^a^	32 637	9.30

**Shielding**		
Non-shielding	337 758	96.24
Shielding	13 208	3.76

a Defined as patients with severe mental illness (based on Quality and Outcomes Framework (QOF) business rules^19^) OR patients with depression (based on QOF rules^19^) OR patient prescribed antidepressants in the last 3 months (excluding tricyclics, as these are commonly used for non-mental health conditions).

GP-to-patient SMS messages increased 3.1-fold, and nurse-to-patient 4.8-fold in April to July 2020 compared to 2019. In April to July 2019, 33% of SMS were sent on the same day the patient had a consultation. By April to July 2020 this had increased to 65% (see Supplementary Figure S1).

Table 2 shows changes from April to July 2019 to April to July 2020. There was an 11% reduction in GP consultations (IRR 0.89) and a 17% reduction for nurses/paramedics (IRR 0.83). GPs did almost three times more remote consultations than the previous year and nurses over five times more (IRR 2.76 and IRR 5.51, respectively). GP in-person consultations dropped to 16% of the previous year and nurses to just over 50% (IRR 0.16 and IRR 0.54, respectively).

**Table 2.** Changes in consulting rates in April to July 2020 compared to April to July 2019 overall, and stratified by patient characteristics^a^

	**All consultations**			**Face-to-face and home visits**			**Telephone, video, and e-consultations**		

**GP consultations**	**IRR**	**95% CI**	***P*-value**	**IRR**	**95% CI**	***P*-value**	**IRR**	**95% CI**	***P*-value**
**Change in consultation rates by variable (2020 versus 2019)^b^**	0.89	0.85 to 0.92	<0.001	0.16	0.14 to 0.19	<0.001	2.76	2.33 to 3.27	<0.001

**Age category, years^c^**			<0.001			<0.001			0.421
0–4	0.81	0.73 to 0.88	<0.001	0.24	0.20 to 0.28	<0.001	2.60	2.14 to 3.15	<0.001
5–17	0.65	0.59 to 0.71	<0.001	0.12	0.10 to 0.14	<0.001	2.19	1.81 to 2.65	<0.001
18–49	0.91	0.83 to 1.00	0.041	0.16	0.14 to 0.19	<0.001	2.77	2.30 to 3.34	<0.001
50–69	0.89	0.81 to 0.97	0.008	0.16	0.14 to 0.19	<0.001	2.76	2.29 to 3.33	<0.001
70–84	0.96	0.87 to 1.05	0.324	0.19	0.16 to 0.22	<0.001	2.84	2.35 to 3.42	<0.001
≥85	1.05	0.96 to 1.16	0.265	0.24	0.21 to 0.29	<0.001	2.78	2.30 to 3.36	<0.001

**Mental health status^c^**			<0.001			0.687			0.229
Good mental health	0.84	0.80 to 0.87	<0.001	0.16	0.14 to 0.18	<0.001	2.61	2.25 to 3.03	<0.001
Poor mental health	1.06	1.01 to 1.10	0.022	0.17	0.14 to 0.19	<0.001	2.97	2.56 to 3.44	<0.001

**Shielding status^c^**			<0.001			0.032			0.644
Non shielding	0.86	0.81 to 0.91	<0.001	0.16	0.14 to 0.18	<0.001	2.67	2.29 to 3.11	<0.001
Shielding	1.09	1.03 to 1.16	0.004	0.20	0.17 to 0.23	<0.001	2.81	2.41 to 3.27	<0.001

**Sex^c^**			0.411			0.776			0.718

**IMD quintile^c^**			0.895			0.867			0.940

**Ethnicity^c^**			0.636			0.076			0.108

**Nurse/paramedic consultations**	**IRR**	**95% CI**	***P* -value**	**IRR**	**95% CI**	***P*-value**	**IRR**	**95% CI**	***P*-value**

**Change in consultation rates by variable (2020 versus 2019)^b^**	0.83	0.76 to 0.91	<0.001	0.54	0.49 to 0.59	<0.001	5.51	3.81 to 7.97	<0.001

**Age category, years^c^**			0.003			<0.001			0.835
0–4	1.00	0.85 to 1.17	0.975	0.83	0.70 to 0.98	0.026	5.71	3.98 to 8.18	<0.001
5–17	0.62	0.53 to 0.73	<0.001	0.29	0.25 to 0.35	<0.001	4.71	3.32 to 6.68	<0.001
18–49	0.87	0.74 to 1.02	0.081	0.53	0.45 to 0.62	<0.001	6.66	4.79 to 9.27	<0.001
50–69	0.89	0.76 to 1.04	0.154	0.57	0.48 to 0.67	<0.001	5.74	4.13 to 7.98	<0.001
70–84	0.86	0.74 to 1.01	0.064	0.56	0.48 to 0.66	<0.001	5.54	3.97 to 7.73	<0.001
≥85	0.83	0.71 to 0.97	0.021	0.51	0.44 to 0.61	<0.001	5.51	3.90 to 7.78	<0.001

**Mental health status^c^**			0.017			0.012			0.966
Good mental health	0.83	0.75 to 0.92	<0.001	0.54	0.49 to 0.59	<0.001	5.78	4.24 to 7.87	<0.001
Poor mental health	0.99	0.90 to 1.10	0.890	0.65	0.58 to 0.72	<0.001	5.72	4.17 to 7.86	<0.001

**Shielding status^c^**			0.001			0.001			0.222
Non shielding	0.83	0.73 to 0.94	0.003	0.53	0.46 to 0.60	<0.001	5.28	3.75 to 7.44	<0.001
Shielding	1.15	1.01 to 1.31	0.031	0.73	0.63 to 0.84	<0.001	7.13	4.99 to 10.19	<0.001

**Sex^c^**			0.390			0.873			0.727

**IMD quintile^c^**			0.958			0.926			0.879

**Ethnicity^c^**			0.163			0.111			0.924

a Table 2 shows output from the negative binomial models.

b The overall changes in consulting in 2020 compared to 2019 are presented.

c P*-values for the interaction between consulting year and patient characteristics are presented. Changes in consulting rates are only presented by the different levels of a patient characteristic for characteristics if the interaction* P*-value is* <*0.05 for at least one of the three outcome models. IMD = Index of Multiple Deprivation. IRR = incidence rate ratio.*

These changes were consistent across sex, IMD, and ethnicity groups (interaction *P*-values >0.05 for all outcome models), but differed by patient age, mental health status, and shielding status, for both GPs and nurses (Table 2).

#### Age

There was no significant change in total GP consultation rates in patients aged >70 years, and a decrease in all other age groups, in particular 5 to 17 years (IRR 0.65, *P*<0.001). The reduction in GP in-person consultations was less for patients who were ≥85 years (IRR 0.24, *P*<0.001) and pre-schoolers (IRR 0.24, *P*<0.001) than ages 5 to 84 years (IRR 0.12 to 0.19, all *P*<0.001). Nurses maintained a greater in-person focus on pre-school children (IRR 0.83, *P* = 0.03) with a larger drop in in-person nurse consultations for all other age groups (IRR 0.29 to 0.57, all *P*<0.001).

#### Mental health

Consultation rates in patients with poor baseline mental health increased between April to July 2019 and April to July 2020 for GPs (IRR 1.06, *P* = 0.02) and stayed constant for nurses (IRR 0.99, *P* = 0.89). GP and nurse consultation rates in patients with good mental health decreased (IRR 0.84 and IRR 0.83, respectively, *P*<0.001). People with good mental health had a greater reduction in nurse in-person consultations (IRR 0.54, *P*<0.001) than people with poor mental health (IRR 0.65, *P*<0.001; interaction *P* = 0.01).

#### Shielding

Consultation rates in shielding patients increased in April to July 2020 compared with April to July 2019 for both GPs (IRR 1.09, *P* = 0.004) and nurses (IRR 1.15, *P* = 0.03). Consultation rates for non-shielding patients decreased. In-person consultations reduced more in non-shielding patients than shielding patients (GP IR 0.16 versus 0.20, interaction *P* = 0.03; nurse IRR 0.53 versus 0.73, interaction *P* = 0.001).

### Qualitative findings

#### Summary of changes through the four rounds

**Figure 2.**
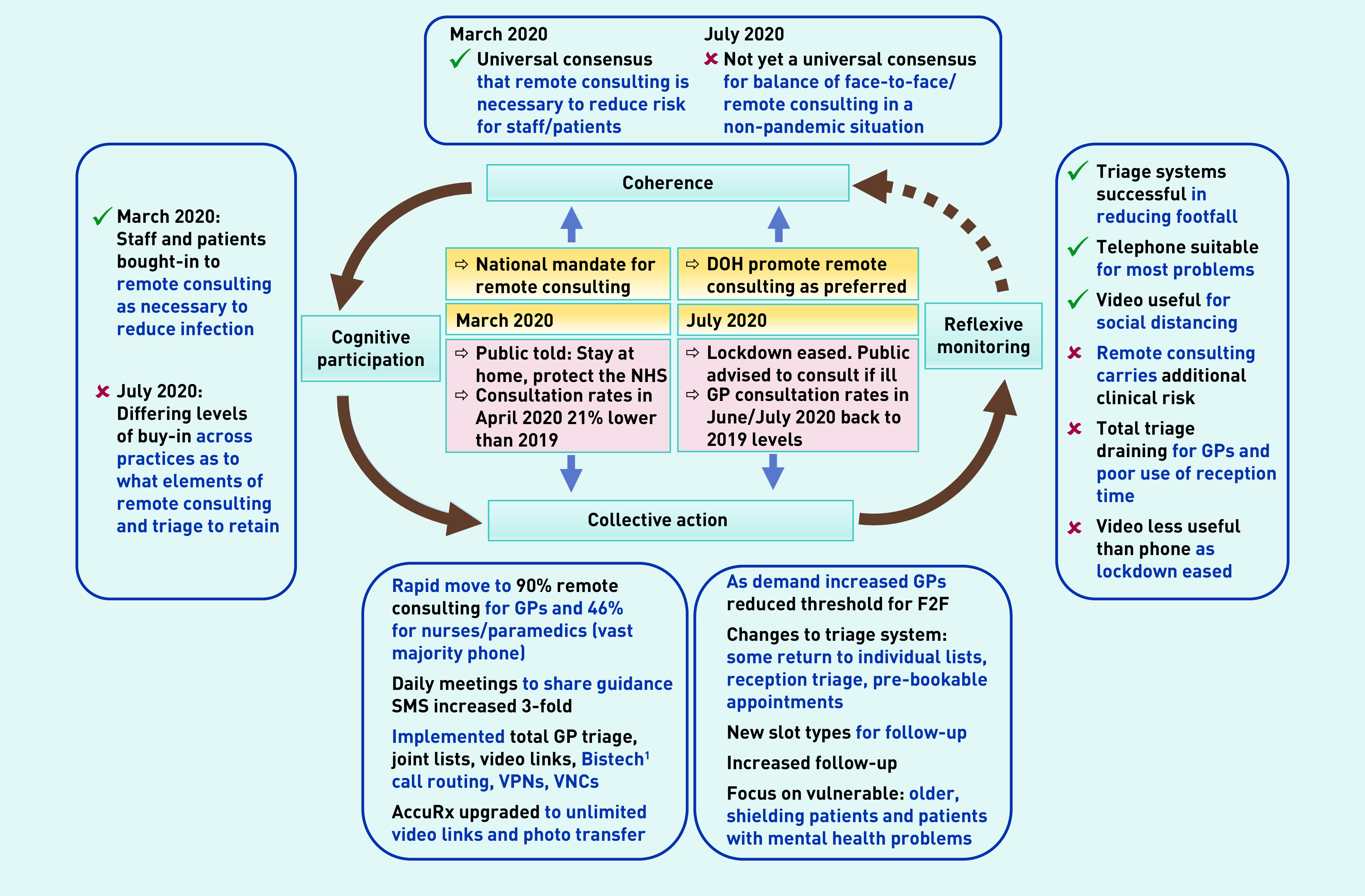
***Normalisation Process Theory model of remote consulting.*** ^1^***Bistech = company that provided telephone call routing to enable some reception staff to work from home or sites closed to patients. DOH = Department of Health. F2F = face-to-face. VPN = Virtual Private Network (provides secure connection to a private network). VNC = Virtual Network Computing (provides remote control of a computer from another location).***

Changes through the four time-periods are described diagrammatically in Figure 2; using Normalisation Process Theory as a framework to explain the implementation of remote consulting.

Participants reported universal consensus on the need for remote consulting and strong buy-in to implementing it in March 2020. In Round 1 (May 2020), practice staff felt a strong sense of achievement in having worked so effectively with partners to implement the necessary changes.^24^ In Round 2, practices were restarting some routine services that were paused in Round 1. This increased volume was challenging because: face-to-face consultations took longer (because of infection control), many telephone consultations required more careful questioning than pre-COVID (because even complex problems were now routinely managed by phone), and video took time to connect.^25^ In Round 3, many participants reported fatigue from holding increased levels of clinical risk, partly because of remote consulting and partly because of the backlog in secondary care.^26^ In Round 4, practices reflected on what to retain post-pandemic. They were keen to avoid a return to unfiltered demand but, despite a suggestion by the Department of Health that remote consulting should be the preferred way of working,^27^ most GPs felt that remote consulting at current levels was unsustainable.^28^

Detailed findings are presented for each of the NPT constructs below.

#### Coherence: making sense of the reasons for remote consulting

Mirroring NHS England advice in March 2020 that face-to-face patient contact should be minimised, Round 1 interviews showed a strong consensus that remote consulting was imperative to protect patients and staff. In later interviews, as UK lockdown eased, this strong coherence reduced, due to lack of clear guidance:

*‘When we started* [March 2020] *it was very clear that your primary goal was to not have people enter this building* […] *whereas now,* [July 2020] *there isn’t any clear NHS England message to guide on your threshold for bringing people in, so I think it’s hard for us to know what we should be doing’.*

(GP, Health centre [HC]20, Round 4)

#### Cognitive participation: buy-in to remote consulting

Round 1 interviews demonstrated universal staff buy-in for rapidly implementing remote consulting:

*‘We’ve had to* [implement remote consulting] *by necessity so again that’s taken out a lot of the onboarding, selling process and they just sort of got on with it like GPs do* .*’*

(GP, HC20, Round 2)

Telephone, video, and SMS were seen as necessary to implement social distancing and as what patients wanted. However, this wholesale buy-in did not apply to e-consultations, which staff perceived as driven by a pre-existing national agenda:

*‘We were told we had to do it* [provide e-consultations] *. There was no motivation at all, apart from the stick* [national policy] ’.

(GP, HC19, Round 4)

Buy-in to remote consulting was strongly tied to the sense of coherence of remote consulting as a current necessity. Clinicians varied as to the extent to which they wanted to continue consulting remotely after the pandemic:

*‘We’re doing it* [move to remote consulting] *because we have to do it, not because it’s how we choose to work.’*

(GP, HC13, Round 1)

‘So certainly, I think the triage by phone and video consulting will be two areas that we will keep but tempered.’

(Practice manager, HC1, Round 1)

#### Collective action: putting remote consulting into action

From March 2020, guidance was regularly issued from various organisations on how services needed to change.^29^^–^^31^ A CCG-led collaboration collated the guidance and sent daily bulletins to practices. Most practices set up small teams to interpret, action, and cascade this within the practice:

*‘Up until this week we were having a daily 9.00 am meeting of a lead doctor, a manager, a receptionist, an IT person and a lead nurse, just to* […] *discuss any Government or CCG, or any other guidelines which had come in overnight, and check on how we were implementing them.’*

(GP, HC10, Round 1)

Practices closed online or walk-in advance consultation booking, implemented same-day total triage, joint GP patient lists, and established infrastructure to allow home working through call routing and remote EMIS access (Figure 2). E-consultation systems were introduced gradually to ensure practices could set-up and embed them properly, and technology for SMS photos and video links was implemented. GPs initially used their own phones and data. As international supply-chain problems eased, the CCG provided laptops, webcams, and improved WiFi. Taking such rapid wholescale action was possible because of the initial drop in demand:

*‘It was very easy to turn around our system from being very face-to-face to telephone* […] *with lockdown the patient demand disappeared for various conditions and so that gave us a bit of room to breathe.’*

(Practice manager, HC18, Round 2)

As demand increased, but COVID-19 cases in BNSSG remained low, practices adjusted systems, opening pre-bookable telephone appointments, reintroducing receptionist triage, and returning to individual GP patient lists.

#### Reflexive monitoring: positive appraisals of remote consulting

Imposing 90% remote consulting created wide recognition that many patients previously seen face-to-face could be safely consulted by telephone. Furthermore, information gathered through triage meant necessary face-to face time was more *‘focused and productive’* (GP, HC18, Round 2).

Some clinicians, previously resistant to telephone consulting, recognised that it was a skill which could improve with practice. Nurses found that telephone consulting worked well for chronic condition reviews, prioritising poorly controlled patients, and seeing patients face-to-face for physical aspects only. Telephone consulting gave GPs greater control of their working day and meant they could type and check information without the patient feeling that they were not listening. GPs noted that patients come to the point more quickly and raise fewer problems by phone:

‘I hope we’ll never go back to just whole mornings of patients booking by themselves, quite often when they don’t need to see a doctor, when it could have been dealt with in another way or by another person.’

(GP, HC9, Round 2)

Video consultation proved useful for dynamic assessment (such as gait and respiratory monitoring) and were particularly useful with children, to assess them visually and reassure the parent:

*‘The* [verbal] *description doesn’t always match up with the clinical picture and being able to actually have a look, that’s very helpful’.*

(GP, HC3, Round 1)

Nurses used video consultations to train patients and/or carers on wound care or administration of injectable long-acting reversible contraception. GPs used video consultations to connect with older people or vulnerable patients in nursing homes or when they were with an allied health professional.

Clinicians used accuRx to send information to patients via SMS before and after a consultation:

*‘I’ll write quite detailed texts to patients who I’ve just spoken to, saying, “You might want to try this website”* […] *all you have to do is cut-and-paste a link and some people then have immediately got the website on their phone.’*

(GP, HC19, Round 2)

SMS proved useful for fitness-to-work notes, contacting patients about prescriptions, and sending questionnaires to risk-stratify people with long-term conditions. Most GPs preferred a photograph-plus-telephone-consultation to video consultations for static problems that require visual assessment (for example, a rash):

*‘Rather than initially setting up a video consultation* [it’s better] *to ask them to take a picture of it* […] *because the patient spends time getting a decent photo, and you’re not hanging on for each video consult for 5 or 10 minutes while you get the technology working.’*

(GP, HC11, Round 1)

Most felt it was too early to appraise the impact of e-consultations. Some practices hoped that the response-window of e-consultations (for example, 48 hours) would enable them to spread demand more flexibly.

#### Reflexive monitoring: challenges with remote consulting

From June to July 2020, as consultation volumes and complexity increased, GPs found telephone consulting at high volumes to be more mentally intense and less satisfying:

‘Working from a long screen of lots of telephone calls, with holding lots of risks for a long time, and having then also removed what many GPs find the most enjoyable part of their job — talking and touching and sensing patients in the room — the day job has become a bit of a hard grind.’

(GP, HC20, Round 2)

Some felt an increased strain in making clinical decisions, prescribing, and holding more clinical risk over the phone:

*‘I had someone* [on the phone] *with a bit of abdominal pain, chest tightness, anxious, pain in feet, PR* [per rectum] *bleeding, you just think “Gosh — where do I even start with this.” Yes, It can be a bit tricky over the phone*.*’*

(GP, H16, Round 4)

Most GPs felt that, although they were seeing patients face-to-face when necessary, in the context of a pandemic, this depended on weighing up competing risks to the patient and practice. Practices with a large older, deprived, or immigrant population pointed out that non-verbal cues were more important in some groups of patients than others:

*‘I work in a relatively deprived multi-ethnic area* […] *sometimes it’s more difficult to be able to take a very clear and reliable history over the telephone and be able to make safe management decisions.’*

(GP, HC5, Round 1)

GPs had varying levels of IT problems with video consultations, highlighting that seamless technology is essential for successful implementation. While GPs had high initial expectations of video calls, as the pandemic eased, many felt that face-to-face was increasingly preferable to video for patients who needed visual assessment:

*‘I think the initial excitement about video consulting* […] *there is quite a bit of faff around it and* […] *there is not that much that it adds.* […] *When we first started and absolutely not seeing patients and that was very useful, now I think probably if you needed a video, you might just think I might just see them* [face-to-face] *at this point.’*

(GP, HC20, Round 4)

Other GPs pointed out that they also often needed to examine the patient and visualise close-up:

*‘I kind of thought I would be doing more video by now, but* […] *I’m still doing mostly phone. I think I’m finding things that I want to see. I want to feel more than see, mostly.’*

(GP, HC8, Round 4)

Some clinicians found it challenging to know when to switch to video and were concerned that they may have missed problems in telephone consultations because patients had not reported physical signs.

E-consultations registered by a clinician were <1% of all consultations in July 2020. The algorithm-based platform was introduced slowly as an alternative to telephone access. This allowed practices to pilot and refine the platform, but also meant that e-consultations were seen as an additional stream of work:

*‘It’s like having more than one email account, isn’t it? You have got to check in all different places for incoming stuff* [it’s] *much more efficient to have everything coming into a single point*.*’*

(GP, HC19, Round 4)

GPs also raised concerns about remote consultations that were commonly raised before COVID-19: first, that e-consultations would be used ‘inappropriately’;^32^ second, that all types of remote consultation would lead to *‘double doing’* (GP, HC8, Round 2);^33^ third, that SMS, e-consultations, and video would increase access for those with IT skills, and enforce already existing health inequities.^4^^,^^34^

## DISCUSSION

### Summary

There was widespread consensus in March 2020 that remote consulting was required to contain COVID-19. Collective action was rapidly taken so that 90% of GP and 46% of nurse consultations were delivered remotely in April 2020. Although consultation rates reduced in April to July 2020 compared to April to July 2019, there was no change or increased rates in older patients, shielding patients, and patients with poor mental health. Telephone consulting was sufficient for many patient problems. SMS-messaging increased more than 3-fold, as it was used for photographs, sending video consultation links, and sharing information with patients after the consultation. Video consulting was useful for children, nursing homes, multidisciplinary team meetings, and problems that require dynamic assessment. However, many GPs preferred a photograph-plus-telephone-consultation for many problems that require visual assessment.

After the initial response to the crisis, some GPs found high levels of remote consulting a strain. Many GPs missed face-to-face contact, were concerned about clinical risk, and found it difficult to set a threshold for seeing patients face-to-face as lockdown eased.

### Strengths and limitations

To the authors’ knowledge, this is the first mixed-methods study on the change in UK general practice employing simultaneous longitudinal interviews with staff, and longitudinal analysis of patient records. A large dataset (*N* = 350 966) was used. The main exposure event was UK lockdown combined with the instruction to deliver remote consulting. As this applied across the UK, the changes in face-to-face and remote consulting are likely to be generalisable across England. By using quantitative ‘consultations’ data, the authors overcame the current issues facing NHS digital experimental ‘appointments’ data, whereby the number of GP–patient interactions taking place since April 2020 has been systematically undercounted.^35^ Previous studies have used the ‘consultation type’ to identify whether a consultation is, for example, face-to-face, telephone, or video.The authors found that GPs rarely changed the type, for example, to ‘video’ if they switched from telephone to video during a consultation. By using clinical codes to identify consultation type, telephone consultations were more accurately identified in the current study than relying on the GP-selected type alone, although the authors recognise that they may have misclassified some video consultations.

A key limitation is that, because patient-facing research was paused when the study commenced, patients were not interviewed. Furthermore, there was some self-selection in practice recruitment and the deprivation mix was slightly polarised, represented by the top and the bottom three quintiles. There was a potential for bias in recording of patient characteristics, for example, older patients are more likely to be missing ethnicity.^36^ Nonetheless, apart from ethnicity, the proportions of missing data are low. Lastly, the findings on e-consultations may not be as transferable as the other findings. The algorithm-based digital triage platform was used as an alternative to telephone access. Other platforms with different features and access models (for example, AskmyGP; https://askmygp.uk) may have had different results.

### Comparison with the literature

Previous research found that convenience for patients is a key benefit of telephone, video, and e-consultations.^37^^–^^40^ During the COVID-19 pandemic an additional obvious benefit was the reduction in virus transmission risk. As with previous studies, the authors found that seamless technology is required to make video consultations work^37^^,^^38^ and where GPs have repeated technical problems, they are less likely to engage with video. Pre-pandemic evidence suggested that both telephone and video consultations are safe and efficient.^3^^,^^37^^,^^39^^,^^41^^,^^42^ However, this evidence was collected in patients with stable conditions who are assessed as suitable for remote consultations by a GP. The authors in this study found that, although GPs can do more by telephone than previously thought, they are increasingly concerned about using such a high proportion of telephone and video consultations.

A greater proportion of post-lockdown remote consulting was found in this study compared to the Royal College of General Practitioners research surveillance centre (RSC) study, which found 26% of appointments were face-to-face and 71% by phone.^12^^,^^43^ This may be because the authors in this study more accurately coded consultation type by combining clinical codes with consultation type. The cross-sectional RSC study found more remote consulting among people in IMD quintile 1 (most deprived).^12^ In this current longitudinal study, no significant effect was found of deprivation on the *change* in the volume of telephone or face-to-face consulting between 2019 and 2020.

Other studies on video consultations in outpatients and primary care have shown uptake of 1%–2% despite positive comments from staff and patients.^3^^,^^44^ The current study’s findings suggest that part of the reason for the low proportion in general practice could be the relatively limited usefulness of video consultations over telephone or face-to-face in the majority of circumstances.

### Implications for research and practice

While remote delivery was suitable for many situations, the key driver for the wholescale shift was the need for social distancing. Managing high levels of remote consulting carries greater GP-perceived clinical risk and, post-pandemic, the proportion of face-to-face consultations should increase. Clinicians recognised that certain problems are suitable for telephone, others for video, some for photo-plus-telephone, and some always require face-to-face. Practice-level policies and protocols on this should be implemented to facilitate receptionist navigation.

For sustained use of remote consulting, technology should be simple to use, integrated with existing workflows, and continue to offer a benefit over face-to-face consulting.^40^^,^^45^ Seamless technology is therefore required; e-consultations should be integrated so that they are not perceived as another stream of work and minimising technical problems should increase take-up of video consultations post-pandemic. The integration of accuRx with GP workflows was a key enabler in increasing GP use of SMS to patients for video links and information.

Because of the pause in patient-facing research, the qualitative aspect of this study does not include the patient perspective. There is a need for more patient-oriented research to explore the impact on patient experience of the shift to remote consulting during the COVID-19 pandemic.

There is wide variation in GPs’ confidence with telephone consultation. GPs recognised that telephone/video consulting requires a separate skillset, which can be improved with training and practice. Such training and support now need retrospective implementation following the rapid progress with remote consultation during the COVID-19 pandemic.

Overall, the shift to remote consulting was a successful initial response to the pandemic and focus was maintained on vulnerable patients. However, promotion of it as a preferred consultation process is premature. Although GPs want to continue some of the benefits of remote consulting, the model will need to evolve.
